# Towards Clinical Trial Readiness: Optimization of a Parallel Robot for Lower Limb Rehabilitation

**DOI:** 10.3390/bioengineering13010026

**Published:** 2025-12-26

**Authors:** Paul Tucan, Oana Maria Vanta, Alin Horsia, Ionut Zima, David Mihai Lupu, Calin Vaida, Daniela Jucan, José Machado, Doina Pisla

**Affiliations:** 1 CESTER-Research Center for Industrial Robots Simulation and Testing, Technical University of Cluj-Napoca, 28 Memorandumului Street, 400114 Cluj-Napoca, Romania; paul.tucan@mep.utcluj.ro (P.T.); oana.vanta@elearn.umfcluj.ro (O.M.V.); alin.horsia@mep.utcluj.ro (A.H.); ionut.zima@mep.utcluj.ro (I.Z.); david.lupu@campus.utcluj.ro (D.M.L.); daniela.jucan@mis.utcluj.ro (D.J.); jmachado@dem.uminho.pt (J.M.); 2 European University of Technology, European Union, 28 Memorandumului Street, 400114 Cluj-Napoca, Romania; 3 Neurology I Department, Cluj-Napoca Emergency Clinical County Hospital, 400012 Cluj-Napoca, Romania; 4 Neurology Department, University of Medicine and Pharmacy “Iuliu Hatieganu”, 400012 Cluj-Napoca, Romania; 5 MEtRICs Research Centre, School of Engineering, University of Minho, Campus of Azurém, 4800-058 Guimarães, Portugal; 6 Technical Sciences Academy of Romania, B-dul Dacia 26, 030167 Bucharest, Romania

**Keywords:** parallel robot, robotic-assisted lower limb rehabilitation, clinical trial readiness, clinician feedback, user feedback, fuzzy logic

## Abstract

This study presents the clinical trial readiness and optimization of a parallel robotic system developed for early-stage lower limb rehabilitation of bedridden patients using feedback from healthy users and clinicians. The system combines a parallel hip–knee mechanism with a Bowden cable-driven ankle module, both actuated by servomotors and controlled through a PLC platform. Experimental tests were performed in laboratory conditions with twenty healthy participants (aged 25–45) and ten clinicians, focusing on safety, ergonomics, clinical usability, and comfort through structured questionnaires. The responses were quantified and analyzed using a Mamdani-type fuzzy logic model, allowing subjective feedback to be converted into objective redesign priorities. Safety, torque capacity, and adaptability emerged as the key areas that need improvement. Subsequent mechanical and structural refinements resulted in substantial gains in user comfort, perceived safety, and clinician-reported applicability. The optimized robotic system demonstrates enhanced functionality and improved readiness for clinical evaluation, highlighting the benefit of incorporating fuzzy logic-based feedback into the development of rehabilitation robots.

## 1. Introduction

Lower limb motor impairment is among the most common consequences of neurological conditions such as stroke, spinal cord injury, and traumatic brain injury. Stroke alone affects more than 15 million individuals annually worldwide, and nearly half of survivors experience persistent deficits in gait, balance, and lower limb motor control that limit independence and long-term quality of life [1,2,3]. Traditional physiotherapy continues to be the preferred clinical approach for restoring gait and mobility, yet it is time-limited and strongly dependent on the therapist’s skill and availability [4,5]. Additionally, conventional therapy does not always deliver the high-intensity, task-specific, and repetitive training necessary to promote robust neuroplastic recovery in the early and subacute phases of rehabilitation [6,7,8,9]. In this context, robotic-assisted gait and lower limb rehabilitation technologies have emerged as essential tools for augmenting therapy dose, improving repeatability, and enhancing clinical efficiency. Over the past two decades, various categories of lower limb rehabilitation robots have been introduced, including treadmill-based exoskeletons, overground wearable systems, end-effector gait trainers, hybrid serial–parallel architectures, and fully parallel mechanisms [6,7,8,9,10]. At present, clinical trials report heterogeneous outcomes. While some studies demonstrate functional improvements, others indicate that robotic therapy does not confer superiority over conventional rehabilitation approaches [11,12,13,14,15]. Nevertheless, several studies highlight significant heterogeneity in protocols and conclude that robotic gait training yields superior outcomes primarily when tailored to patient needs and combined with conventional interventions [16,17,18,19,20,21,22,23]. End-effector devices, such as G-EO-type trainers (Reha Technology AG, Olten, Switzerland), also show promising improvements in gait speed and walking capacity, with studies reporting results comparable to exoskeleton-based systems [20,21,24,25]. Meanwhile, wearable robotic exoskeletons (e.g., EksoNR, Indego (Ekso Bionics Holdings Inc., San Rafael/Richmond, California, USA)) have demonstrated feasibility and functional benefits for subacute stroke and spinal cord injury populations, though they remain limited by high cost, extensive setup time, and significant therapist workload [26,27,28,29,30,31,32].

Despite their clinical contributions, commercial exoskeletons and gait trainers are generally optimized for chronic stage rehabilitation and require patients to bear weight and maintain upright posture. However, early rehabilitation, particularly in acute or immediately post-acute contexts, requires controlled mobilization of individual joints, careful passive stretching, and prevention of contracture, often in supine or semi-recumbent positions [33,34,35,36]. Furthermore, the effectiveness of robotic rehabilitation is strongly influenced by patient-specific characteristics, including impairment severity, balance deficits, spasticity level, and anthropometric variability [16,19,21,37]. Guidelines emphasize that robotic systems should complement, rather than replace, therapist-guided therapy and must, therefore, be adaptable, ergonomically safe, and efficient to operate in clinical practice [37,38,39]. Practical drawbacks, such as insufficient torque, limited customization to limb geometry, complex setup procedures, and a lack of clinician-centered interfaces, continue to affect the clinical adoption of such systems [10,32,40].

Parallel robots have attracted increasing attention for early-stage rehabilitation due to their high stiffness, accurate motion control, and superior force transmission properties [15,41]. Several researchers have explored Stewart platforms, cable-driven manipulators, and hybrid parallel–serial structures for passive or active lower limb mobilization [41,42,43,44]. Devices such as LegUp(Research prototype, Technical University of Cluj-Napoca, Cluj-Napoca, Romania) have demonstrated the feasibility of providing multi-joint mobilization for bedridden patients using parallel architectures [34,45]. Recent investigations have optimized these systems using advanced kinematic synthesis, dynamic modeling, and integration with augmented reality or serious games to improve patient engagement and therapeutic outcomes [34,45,46]. Mechanical synthesis techniques, including chord-angle descriptors and optimized five-bar linkages, have further expanded the design space for lower limb rehabilitation robots, enabling more biomimetic and controllable joint trajectories [47].

Despite these advances, an important gap persists. Few rehabilitation robots explicitly integrate structured clinicians and user feedback into the optimization cycle to assess safety, comfort, ease of setup, torque adequacy, adaptability, and ergonomic design, characteristics that play decisive roles in real clinical acceptance.

The aim of this study is to determine the clinical trial readiness of a parallel robot for spatial lower limb rehabilitation of bedridden patients in the acute, post-acute, or chronic phase. The assessment protocol is based on experimental tests performed in laboratory conditions, in a controlled environment under clinicians’ supervision, using healthy subjects. During the experimental tests, the clinicians assess the capability of the robot to perform the medical tasks while the subject of the experiment assesses the comfort of the patient, ergonomics, or other parameters that may influence the patient’s disposition during the rehabilitation. Two types of structured questionnaires are used, one for the health subject and one for the clinician. The experiments were performed on 20 healthy subjects, while feedback from 10 clinicians was obtained. Their feedback, collected through standardized questionnaires, was processed using a Mamdani-type fuzzy logic framework to transform subjective impressions into quantitative design priorities, supporting an iterative and evidence-based refinement of the device’s mechanical configuration, safety enclosures, and calibration mechanisms. Following the readiness assessment, several mechanical improvements were required, and after the enhancement of the robot, experimental tests were repeated, and the readiness for clinical trials was determined again using the same method.

Beyond kinematic and workspace considerations, the present platform introduces elements aligned with early clinical deployment, including tool-less anthropometric fitting in supine positioning, enclosure of moving transmission components for passive-mode safety, and PLC-level emergency stop redundancy. Furthermore, iterative redesign was driven by structured clinician and user feedback through a fuzzy logic prioritization framework, distinguishing this device from laboratory-oriented parallel manipulators and positioning it for controlled feasibility trials.

The paper is structured a follows. The Introduction presents the clinical context of lower limb motor impairment, current challenges in early-stage neurorehabilitation, and the rationale for adopting a parallel robotic architecture. It also highlights the need for integrating structured clinician and user feedback into the development of rehabilitation technologies. The Section 2 details the architecture of the LegUp robot, including its mechanical and control subsystems, the medical rehabilitation protocol, the participant cohorts, and the experimental evaluation procedures. This section also describes the fuzzy logic framework used to transform qualitative assessments into quantitative design priorities. The Section 3 presents the findings obtained from the healthy participants and clinicians, including descriptive statistics, fuzzy priority indices, and correlation analyses that reveal the dominant factors influencing clinical readiness. The Discussion interprets these findings in the broader context of rehabilitation robotics, explains how the identified limitations informed engineering redesigns, and reflects on the methodological strengths and constraints of using fuzzy inference systems for user-based optimization. Finally, the Conclusion summarizes the key contributions of the study, outlines the improvements achieved through feedback-driven refinement, and identifies the next steps required for transitioning the LegUp platform toward patient-centered clinical trials.

## 2. Materials and Methods

### 2.1. Parallel Robot Architecture

**Figure 1:**
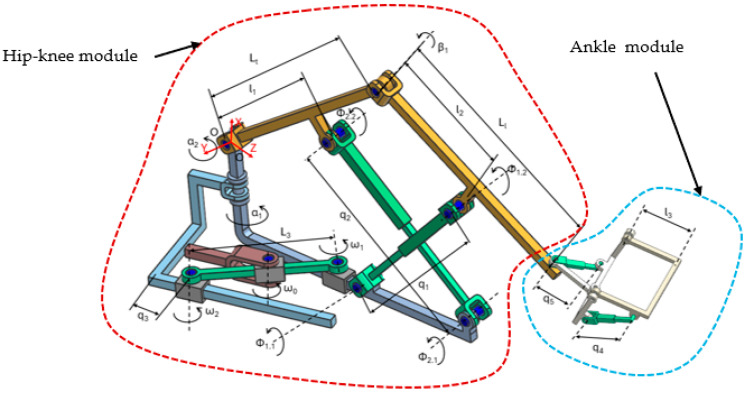
The kinematic scheme of LegUp [48,49].

The LegUp parallel robot for lower limb rehabilitation of bedridden patients is composed of two main modules: the hip–knee module and the ankle module (Figure 1). The two modules are joined in the lower tibia area in such a manner that the ankle of the patient fits the motion center of the ankle module. The hip–knee module is actuated using 3 prismatic active joints: q_1_ and q_2_ joints are used to create the combined flexion and extension motion of the hip and knee. To help generate this motion, the flexion-extension hip–knee mechanism has an additional 6 passive revolute joints (ϕ_1.1_, ϕ_1.2_, ϕ_2.1_, ϕ_2.2_, β_1_, and α_2_); the q_3_ joint actuates the mechanism for the adduction and abduction of the hip. The mechanism also contains 4 passive revolute joints (ω_1_, ω_2_, ω_3_, and α_1_) in order to generate the adduction and abduction motion of the hip joint. The ankle module uses two prismatic joints actuated via Bowden cables; the q_4_ joint generates the plantar flexion and the dorsiflexion of the ankle, while the q_5_ joint generates the inversion and eversion motion.

**Figure 2:**
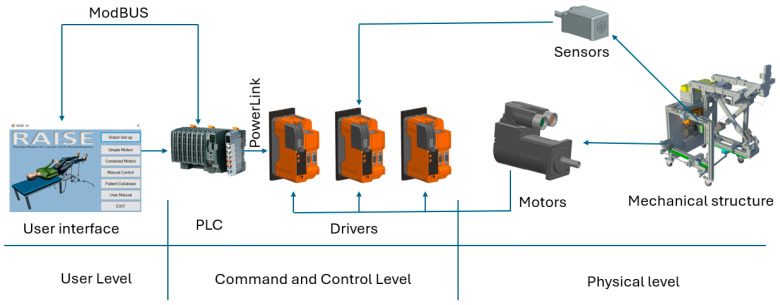
Hardware configuration of the LegUp robot control system.

**Figure 3:**
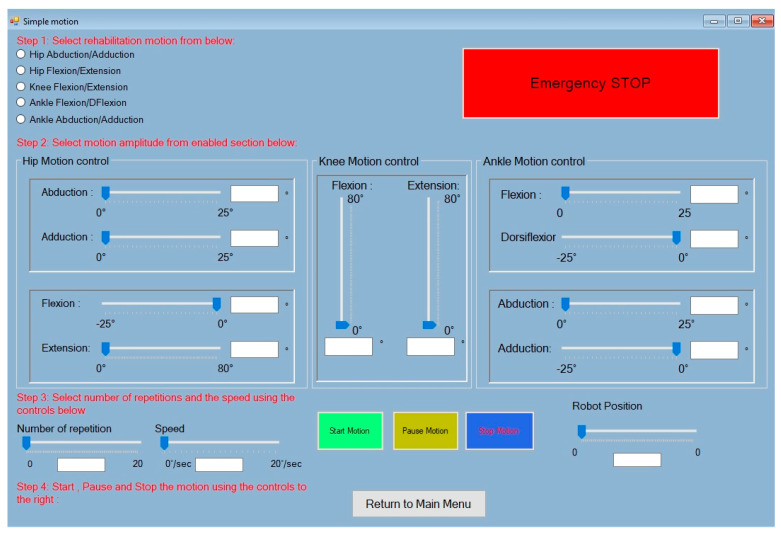
User interface of the LegUp robot.

The mathematical modeling of the robot was previously presented in [47], while an improved control method for smoothing the robot trajectory during the rehabilitation procedure was presented in [48]. The hardware configuration (see Figure 2) of the robot is divided into 3 levels, including the User Level, containing a Visual Studio 2019 [49] interface developed as simply as possible for the medical personnel (see Figure 3), which contains the possibility to perform isolated rehabilitation motions of each joint and input motion amplitudes, number of repetitions, and the speed for each motion.

The Command and Control Level contains the PLC and the drivers for actuating the motors of the robot. The PLC communicates with the user interface, which runs on a local computer, via the ModBus [50] protocol, and the communication between the PLC and the drivers is made via Powerlink [51]. Each driver is able to control two servomotors and receive an input signal from two sensors. The Physical Level of the hardware architecture contains the mechanical structure of the robot, the servomotors, and the sensors used for robot initialization.

### 2.2. Robotic-Assisted Medical Protocol

**Figure 4:**
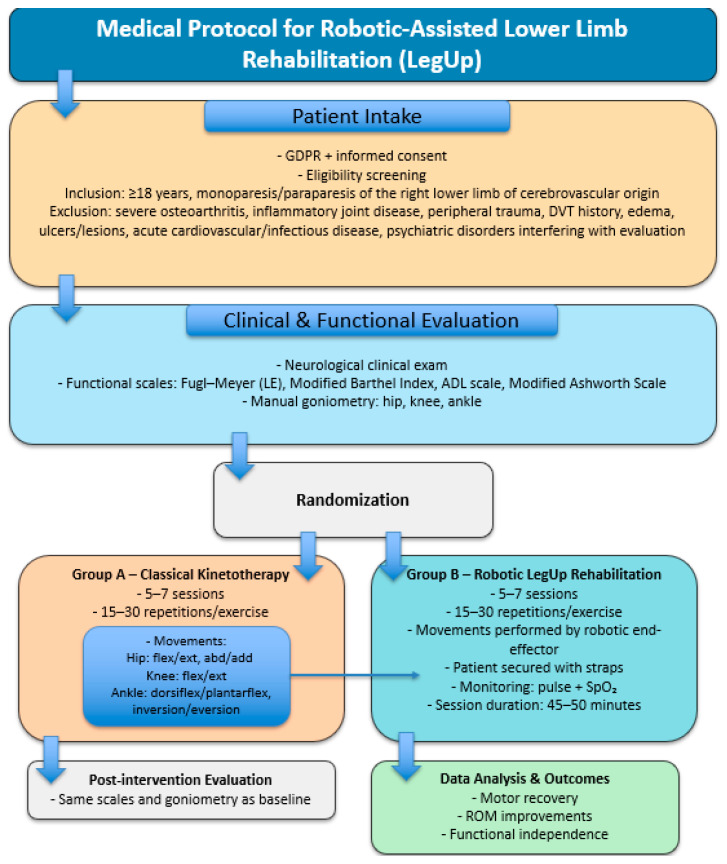
Medical protocol for robotic-assisted lower limb rehabilitation.

In order to prepare the robot for the clinical trials, a medical rehabilitation protocol was developed with the help of the medical rehabilitation experts. A schematic representation of the protocol is presented in Figure 4.

The rehabilitation protocol using the LegUP robotic system is designed to evaluate and enhance motor recovery in adults with monoparesis or paraparesis of the right lower limb of cerebrovascular origin. Eligible participants are first informed about the study objectives and procedures, after which GDPR-compliant informed consent is obtained. Inclusion criteria include patients aged 18 years or older who present with lower limb motor deficits due to ischemic or hemorrhagic stroke, while exclusion criteria eliminate those with severe osteoarthritis, active inflammatory joint disease, peripheral trauma, a history of deep-vein thrombosis, limb edema, skin lesions, acute cardiovascular or infectious conditions, or psychiatric disorders that may interfere with cooperation or outcome evaluation. Once eligibility is confirmed, patients receive a coded identifier to ensure confidentiality. Before starting the intervention, all participants undergo a standardized clinical and functional evaluation performed jointly by a neurologist and a physiotherapist. This evaluation includes a complete neurological examination and several validated functional scales: the Fugl–Meyer Assessment [52] for the lower extremity to quantify motor impairment, the Modified Barthel Index [53] to assess daily functional independence, the ADL scale [54], and the Modified Ashworth Scale [55] to evaluate muscle tone. Additionally, joint mobility is measured using manual goniometry at the hip, knee, and ankle. These baseline measurements are used as reference points for post-intervention comparison and to tailor the therapeutic plan to each patient’s physical status.

Outcome evaluations (Fugl–Meyer, Modified Barthel Index, ADL, and Modified Ashworth Scale) will be administered by clinical personnel not involved in therapy delivery, and assessors will be blinded to group allocation whenever feasible. Standardized scoring guidelines and repeated-rater consistency will be employed to minimize inter-rater variability.

Following the initial assessment, patients are randomly assigned to either conventional physiotherapy or robotic-assisted rehabilitation using the LegUP system. Both groups complete a series of five to seven daily sessions, allowing for consistent exposure to therapeutic activity. Patients allocated to classical kinetotherapy follow a structured program of passive and active-assisted exercises that target recovery of joint mobility in a proximal-to-distal pattern. The physiotherapist manually guides the patient’s limb through repetitions of hip flexion–extension and abduction–adduction, knee flexion–extension, and ankle dorsiflexion–plantarflexion with inversion–eversion. Each exercise is performed for 15 to 30 repetitions per session, with progression determined by the patient’s tolerance and neuromuscular response.

Randomization in the planned clinical study will be stratified by baseline impairment level (e.g., Fugl–Meyer lower limb score) and time from onset, thereby reducing initial functional imbalance between arms. Both groups will receive an equivalent therapy dose (5–7 daily sessions of approximately 45–50 min) with matched joint mobilization content and repetition volume. Conventional physiotherapy will follow a structured protocol designed to mirror the robotic lower limb sequence, thereby controlling for session intensity.

Participants in the robotic rehabilitation group perform the same categories of lower limb movements, but these are delivered through the LegUP robotic system. The affected limb is secured to an end-effector capable of generating controlled and repeatable trajectories that replicate the natural joint movements used in manual therapy. Each session lasts approximately 45 to 50 min and includes 15 to 30 repetitions per movement, with adjustments managed by the supervising therapist. Throughout the robotic-assisted sessions, patient safety is ensured by continuous monitoring of pulse and peripheral oxygen saturation. The robotic system provides consistent movement parameters and reduces inter-therapist variability, offering a highly standardized method of delivering repetitive motor training.

Concomitant treatments (pharmacological or nursing) will follow standard clinical practice and will be recorded for both groups so that potential imbalances can be adjusted for during analysis (e.g., ANCOVA with baseline covariates).

At the end of the therapy period, all initial clinical and functional assessments are repeated. The post-intervention results are directly compared with the baseline findings, enabling the evaluation of motor progress, joint mobility improvements, functional independence, and changes in muscle tone. This comparison also permits an assessment of the relative effectiveness of robotic-assisted therapy compared to classical physiotherapy and provides valuable information for future optimization of the LegUP system’s rehabilitation protocols.

### 2.3. Mechanical Design of the LegUp Robot

**Figure 5:**
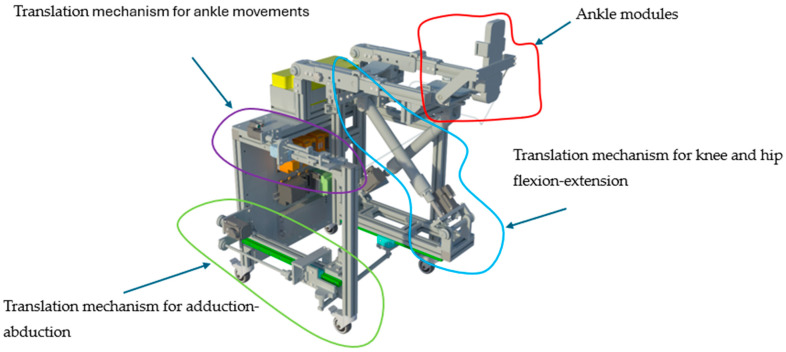
The 3D model of LegUp.

Figure 5 presents the virtual model of the LegUp robot. Positioned at the upper section of the device, the ankle module is responsible for securing the patient’s foot and delivering stabilized mobilization across the distal joint. This dedicated module, highlighted in the figure, ensures accurate transmission of robotic trajectories to the ankle complex. Its placement allows the LegUp system to guide dorsiflexion, plantarflexion, inversion, and eversion with high fidelity, minimizing compensatory movements and ensuring that forces applied to the limb remain well-controlled and anatomically consistent. The rigidity and adjustability of the ankle holding structure accommodate varying patient anthropometrics while ensuring alignment of rotational axes with the patient’s ankle joint. Proximal to the ankle assembly, the system incorporates a Bowden cable translation mechanism for ankle movements, responsible for generating the controlled linear trajectories that enable the robotic execution of the prescribed ankle exercises. By isolating this translation unit, the LegUp system enhances the precision and repeatability of ankle mobilization, enabling therapists to prescribe controlled movement amplitudes, velocities, and repetition counts. This component also plays a key role in ensuring that motion patterns mimic natural physiological kinematics, which is critical in neuroplasticity-based rehabilitation. The central portion of the system houses the translation mechanism for knee and hip flexion–extension, which is one of the primary structural components. This mechanism drives the sagittal plane mobilization of the leg, enabling robotic assistance for two of the most functionally relevant motions in gait and mobility training: knee bending and hip flexion–extension. The geometry of this mechanism allows coordinated movement of both joints while maintaining stable limb support, reducing therapist workload, and ensuring consistent application of therapeutic trajectories. The module’s design supports both passive and active-assisted modes, enabling customized rehabilitation strategies depending on patient strength and motor control. In parallel to these components, the lower region of the device includes the translation mechanism for hip adduction–abduction, visualized in the figure as a long horizontal rail structure. This module enables controlled coronal-plane motions, essential for pelvis stabilization, weight-shifting, and restoration of lateral balance reactions. The capability to reproduce hip adduction–abduction is not commonly present in commercial rehabilitation robots, positioning LegUp as a more comprehensive system capable of addressing multi-planar deficits typical after stroke or traumatic neurological injury. The integration of these four translation mechanisms allows the LegUp system to sequentially or simultaneously guide a complex combination of lower limb movements. By distributing mechanical tasks across multiple independent modules, the architecture increases workspace flexibility, reduces mechanical interference, and improves patient safety by allowing each degree of freedom to be finely regulated. The mechanical design further includes support frames, adjustable linkages, and linear actuators that jointly ensure stability, patient adaptability, and robustness during repeated therapeutic cycles. This design approach allows clinicians to implement personalized, repeatable, and precisely controlled motor training protocols targeting the hip, knee, and ankle joints with high accuracy and reliability.

### 2.4. Experimental Protocol and Participants

After the robot prototype was ready, functional experiments were conducted in a controlled laboratory setting on healthy adult volunteers and a group of experienced rehabilitation clinicians. The medical protocol defined in Section 2.3 was adapted with the specification that the subjects were healthy persons, so no baseline clinical assessment was required, and the ethical approval was waived; the subjects were required to sign a formal consent. All procedures conformed to institutional and ethical standards for non-patient human subject testing. The user cohort included twenty healthy adults with no history of significant neurological or orthopedic disease. Ten of them had prior exposure to robotic rehabilitation devices, and ten were naive users. The clinician cohort consisted of ten rehabilitation professionals, including neurologists and physical therapists, with a minimum of five years of clinical practice in neurorehabilitation [56].

Each user participant underwent a standardized session in which they were positioned in the robot in supine posture. The hip–knee module executed a sequence of flexion and extension movements at a range between 0 and 100 degrees, followed by hip abduction–adduction with a degree of motion between 0 and 30 degrees. The ankle module performed sequences of dorsiflexion–plantar flexion with a range of -30 degrees and 30 degrees and inversion–eversion with amplitudes between −10 and 10 degrees, which were chosen to be comfortable while covering a significant portion of the normal passive range of motion. All movements were purely passive, with the robot driving the limb. Clinicians attended and supervised the sessions and could interrupt the robot at any time via the PC interface or emergency stop.

At the end of each session, user participants completed a structured questionnaire tailored to their subjective experience. The questionnaire contained items assessing ease of setup and operation, comfort during the session, presence or absence of pain or discomfort, perception of safety, physical fatigue, naturalness of limb movements, perceived usefulness of the ankle module, overall stability, motivation, and global satisfaction. Responses were recorded predominantly in ordinal categories (for example, very comfortable, comfortable, neutral, uncomfortable, and very uncomfortable or no pain, mild discomfort, moderate pain, and severe pain) and for global satisfaction on a 1–5 scale. To minimize subjective bias, participants were evenly divided between individuals with prior exposure to lower limb rehabilitation robotics (*n* = 10) and naïve users (*n* = 10). All individuals received identical standardized instructions and were evaluated in the same laboratory environment immediately after the experimental session. Clinicians completed their questionnaires independently of participants, ensuring that expert impressions were not influenced by end-user responses. Open-ended questions asked participants to describe what they liked most about the robot, what they found difficult or uncomfortable, and what they would improve.

Clinicians completed a separate but parallel questionnaire focusing on system setup, perceived safety, relevance to rehabilitation goals, adaptability to different patient morphologies and impairment levels, observed patient comfort, impact on their own workload, software interface usability, and integration of the robotic session into standard therapy. They also rated item responses on ordinal scales (for example, excellent, good, average, poor, very poor) and provided free-text comments. All clinician assessors had ≥5 years of neurological rehabilitation experience. Their more conservative scoring of torque sufficiency and adaptability, compared with healthy users, suggests that the data collection procedure did not induce systematic positive bias. The user questionnaire is presented in Table 1, while the clinician’s is presented in Table 2. (Detailed information referring to user inputs are provided in Appendix A Table A1, and regarding the clinicians input in Appendix A Table A2, respectively)

The raw data consists of categorical responses for each item and participant. For statistical analysis, ordinal categories were mapped to numerical scores according to monotonic scales. This mapping allowed the calculation of descriptive statistics, such as means and standard deviations, across participants for each item. For a generic item *j* with *N* respondents and numerical responses *x_i,j_*, the mean and standard deviation are defined as follows:

(1) $$\mu_{j}=\frac{1}{N}\sum_{i=1}^{N}x_{i,j}$$

(2) $$\sigma_{j}=\sqrt{\frac{1}{N}\sum_{i=1}^{N}{\left(x_{i,j}-\mu_{j}\right)}^{2}}$$

To obtain composite indicators that weigh clinical relevance more heavily than naive user comfort, a weighted combined score *S_j_* was defined as follows:

(3) $$S_{j}=w_{c}\cdot \mu_{c,j}+w_{u}\cdot \mu_{u,j}$$

where $\mu_{c,j}$ and $\mu_{u,j}$ are the mean scores for clinicians and users on the same conceptual dimension and *w_c_* and *w_u_* are weights satisfying *w_c_* + *w_u_* = 1. In this study, weights were chosen as *w_c_* = 0.65 and *w_u_* = 0.35, reflecting the higher priority of clinical safety and relevance in design decisions.

The weighting scheme used to compute the combined clinician–user score (Equation (3)) was validated through face validity checks comparing three parallel rankings: clinician scores alone, user scores alone, and the weighted aggregate. The domains that clinicians regarded as clinically critical (torque sufficiency and adaptability) remained the lowest scoring across all three rankings, indicating that the weighting scheme emphasizes professional safety concerns without suppressing user feedback.

Because no validated assessment tool exists for the usability of supine passive mobilization manipulators, the questionnaires were developed ad hoc, drawing on constructs recurrent in validated exoskeleton usability research (comfort, perceived safety, trust, alignment burden, and clinical workload). Internal coherence was indirectly supported by convergence between Likert trends and open-text responses and by systematic differences between clinicians and naïve participants.

### 2.5. Fuzzy Logic Model for Feedback-Based Optimization

To transform the heterogeneous human feedback into quantifiable design priorities, a fuzzy logic evaluation framework was developed. This approach was selected due to its capacity to handle the inherent subjectivity, uncertainty, and linguistic nature of human perceptions captured in questionnaires, which are difficult to model using traditional binary or statistical methods alone [57]. The input variables to the fuzzy system were defined as five key criteria derived from the consolidated questionnaire responses: user comfort, perceived safety, adaptability to patients, torque sufficiency, and setup and calibration effort. Each input variable was defined on a normalized scale from 0 to 1, obtained by linearly mapping the corresponding weighted combined score, *S_j_*, from its original numerical response range (e.g., 1 to 5). To model the subjective interpretation of these scores, three linguistic labels were defined for each input: low, medium, and high. These labels were characterized using triangular membership functions, $\mu_{low}(S)$, $\mu_{med}(S)$, and $\mu_{high}(S)$, which assign a degree of membership between 0 and 1 to any given normalized score [52]. Triangular membership functions were selected due to their linguistic interpretability for clinician-driven decision-making and their smooth overlap across low–medium–high regions. The redesign threshold *P* ≥ 0.70 corresponds to the region in which the centroid of the aggregated output is dominated by high or very high membership functions. Sensitivity checks performed in the 0.60–0.80 range did not alter domain ranking, indicating robust prioritization. This fuzzification step is crucial for the optimization process, as it translates crisp numerical scores into a flexible linguistic framework. This allows the subsequent inference engine to reason with imprecise inputs and apply expert-derived rules that reflect the complex, non-linear relationships between patient/clinician satisfaction and engineering design priorities.

(4) $$\mu_{low}(S)=\max\left(0,\frac{0.5-S}{0.5}\right)$$

(5) $$\mu_{med}(S)=\max\left(0,1-\frac{\left|0.5-S\right|}{0.5}\right)$$

(6) $$\mu_{high}(S)=\max\left(0,\frac{S-0.5}{0.5}\right)$$

In the equation, there is S∈[0,1]. These functions assign graded truth values to linguistic assessments, such as “comfort is high” or “adaptability is low.”

The output variable of the fuzzy system is the design priority index *P*, representing the urgency and magnitude of required improvements for each criterion. The output space is partitioned into linguistic labels of low, medium, high, and very high, with overlapping triangular membership functions defined analogously. Expert-derived rules capture the relationship between input assessments and output priority; for example, “IF safety is low THEN priority is very high,” “IF adaptability is low AND torque is medium THEN priority is high,” and “IF comfort is high AND safety is high THEN priority is low.”

To ensure the internal validity of the fuzzy inference system, the linguistic rule base was generated jointly by rehabilitation clinicians and robotic engineers and was benchmarked against a set of synthetic “stress-test” input configurations. These scenarios reflected clinically intuitive cases (e.g., very low perceived safety with otherwise favorable comfort and adaptability, or high torque sufficiency combined with low adaptability), and the resulting priority index was verified to align with the expected expert ranking. The broad overlap of triangular membership functions was selected intentionally to avoid abrupt changes in the priority score in response to small variations in user input, thus improving robustness.

The results of the fuzzy inference are presented in Appendix A Table A3.

The fuzzy inference engine uses standard min–max composition (Mamdani-type inference). For each rule, the degree of activation is computed as the minimum of the involved input memberships, and the output membership functions are clipped accordingly. The aggregated fuzzy output membership function $\mu_{P}(p)$ over the priority domain *p* is obtained as the maximum of all rule outputs.

To obtain a crisp priority index *P* for decision-making, centroid defuzzification is applied [53] as follows:

(7) $$P=\frac{\int_{0}^{1}p\cdot \mu_{p}(p)dp}{\int_{0}^{1}\mu_{p}(p)dp}$$

A threshold of P≥0.70 was used as a criterion to flag any dimension as requiring redesign, while values below 0.4 were interpreted as low priority and values between 0.4 and 0.7 as moderate.

The threshold for critical redesign (*P* ≥ 0.70) corresponds to the region in which the “high” and “very high” output membership functions dominate the aggregated fuzzy set. In practice, this band reflects conditions in which the system assigns a strong belief that redesign is required, whereas values between 0.40 and 0.70 indicate moderate redesign interest, and *P* < 0.40 represents low priority. Informal sensitivity checks showed that modest variations in the threshold (±0.05–0.10) did not change the identity of domains classified as critical.

**Figure 6:**
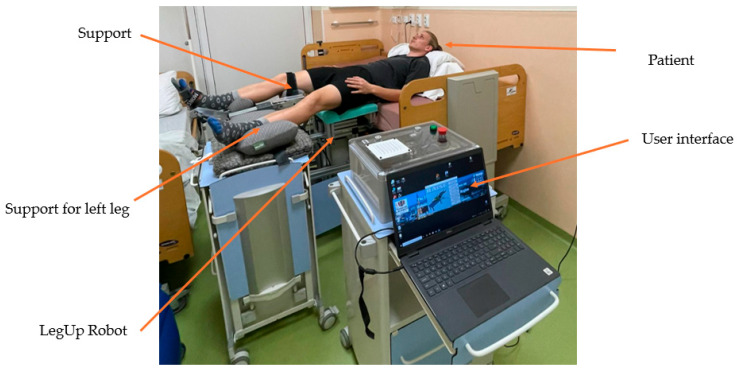
Experimental test setup.

Figure 6 illustrates the experimental setup during the laboratory tests. A height-adjustable hospital bed was used for the subject, which was placed with its back on the bed and tip on the rehabilitation robot. An adjustable support was used for the subject to comfortably place its left leg during the rehabilitation. The user interface ran on a laptop placed near the rehabilitation bed.

## 3. Results

The analysis of the questionnaire responses collected from twenty healthy participants aged 25–45 years and ten experienced rehabilitation clinicians revealed a coherent and interpretable structure of user–robot interaction quality. Most participants rated the robot as comfortable, safe, and clinically meaningful while also identifying specific domains where mechanical and operational improvements were necessary. The clinicians provided more critical insights than the healthy users, particularly regarding torque sufficiency and adaptability, reflecting their clinical experience with neurologically impaired populations.

When user and clinician data were combined using the weighting strategy described earlier, comfort and perceived safety achieved the highest scores, whereas torque sufficiency, adaptability, and setup effort appeared as the more sensitive domains. These distributions form the basis for subsequent fuzzy analysis.

**Figure 7:**
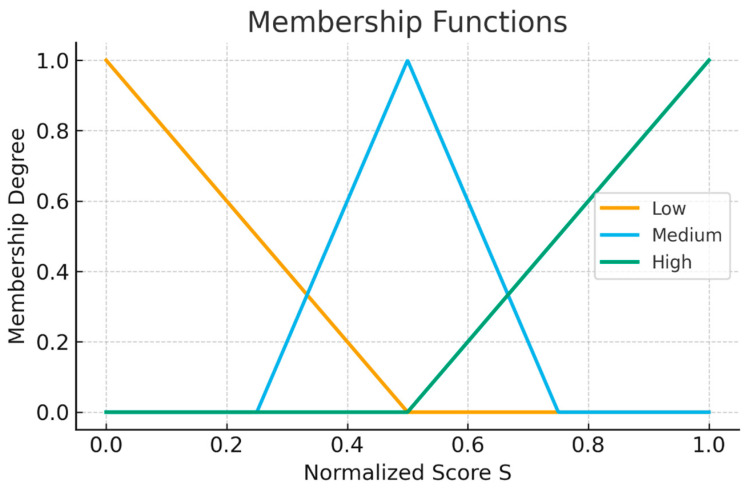
Membership functions used for fuzzification.

All normalized combined scores were mapped through the triangular membership functions for the linguistic variables of low, medium, and high. These functions serve as the foundation for converting numeric input scores into fuzzy membership degrees. As shown in Figure 7, the low membership function peaks at *S* = 0, the medium membership function peaks sharply at *S* = 0.5, and the high membership function peaks at *S* = 1. This allows the fuzzy system to interpret intermediate values with nuance, particularly for dimensions where subjective evaluations exhibited moderate variability across participants. To provide a clear quantitative analysis of the feedback received, the mean and standard deviation were calculated for each item of the questionnaires administered to both clinicians and users. These values, presented in Table 3, offer a statistical basis for evaluating the perception of the rehabilitated robot and serve as input for the fuzzy logic analysis.

The shape of these membership functions ensures that moderate scores (e.g., safety scores around 0.6–0.7) contribute partially to both medium and high fuzzy sets, which becomes important for composite rules involving clinician-prioritized variables, such as safety and torque.

After fuzzification, the Mamdani inference engine evaluated each dimension across all rules, aggregating the resulting outputs and defuzzifying them into crisp priority values. The resulting priority scores revealed a clear separation between domains that require immediate mechanical intervention and those that are already acceptable for clinical progression.

**Figure 8:**
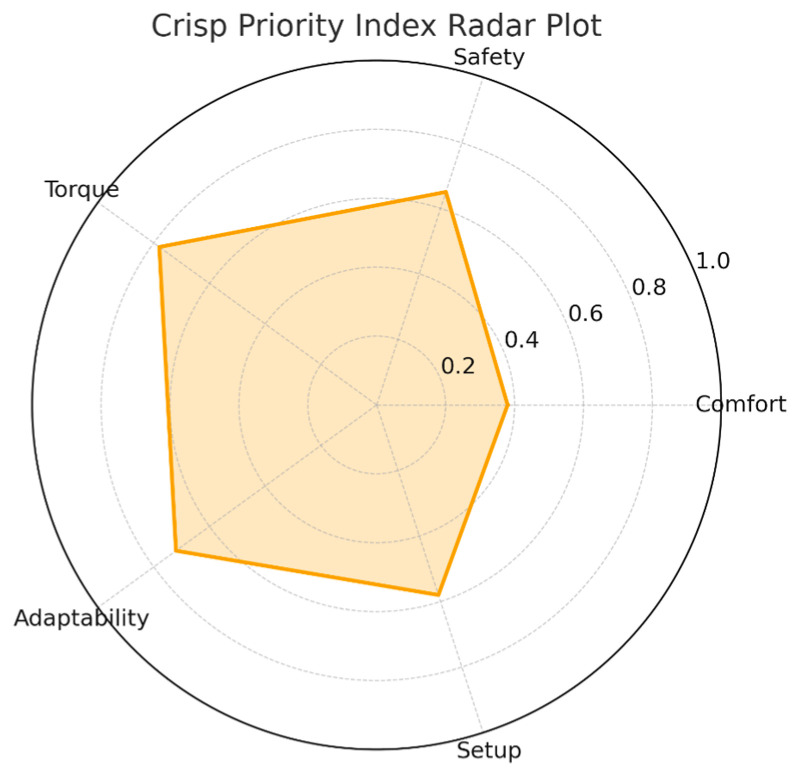
Crisp fuzzy priority index radar plot.

The radar plot in Figure 8 presents the crisp priority indices for all five primary evaluation dimensions. Torque sufficiency exhibited the strongest need for redesign, followed closely by adaptability. Safety fell into an intermediate revision zone, reflecting clinician concerns about mechanical coverings and visibility of moving components. Setup effort scored moderately, whereas comfort was far below the redesign threshold.

The dominance of torque and adaptability in this radar representation is consistent with the clinicians’ written feedback. They anticipated that patients with spasticity or decreased joint compliance would require greater mechanical strength and more intuitive limb alignment adjustments. This fuzzy-driven prioritization thus directly formalizes the qualitative observations into algorithmically derived optimization guidance.

**Figure 9:**
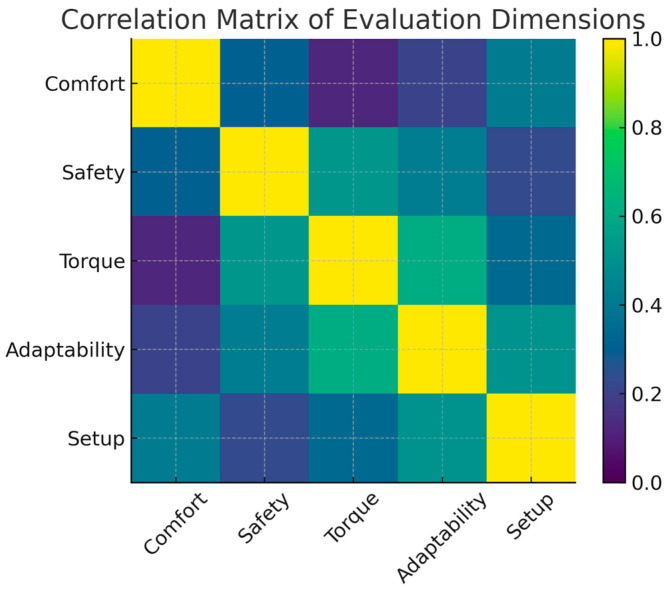
Correlation matrix of evaluation dimensions.

To further explore the relationships among the evaluation categories, a correlation matrix of the combined scores was generated. The heatmap shown in Figure 9 reflects how participants’ perceptions of one system property co-vary with others.

The matrix shows the following.

Torque sufficiency correlates strongly with adaptability (r ≈ 0.61). Clinicians who perceived insufficient torque also tended to report limited adaptability to different patient body types.

Comfort correlates weakly with torque (r ≈ 0.12), suggesting that comfort is largely independent of actuator strength because movements were passive and controlled.

Safety correlates moderately with comfort (r ≈ 0.31), indicating that perceived safety is partly influenced by the sensation of stability and smoothness during movement.

Setup effort correlates significantly with adaptability (r ≈ 0.51), reflecting the mechanical and ergonomic adjustments required during initial positioning.

These correlations help validate the structure of participant responses and support the interpretation that mechanical redesign should focus primarily on torque and adaptability.

**Figure 10:**
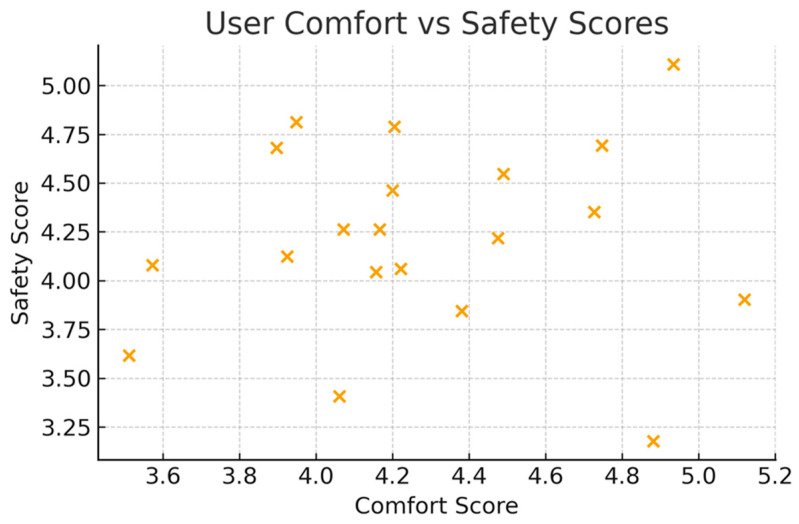
User comfort vs. safety scores.

Beyond global metrics, it is informative to examine finer-grained interactions among user-based indicators. Figure 10 shows the scatter relationship between comfort and safety scores among healthy subjects. Although individual responses vary due to personal sensitivity to movement and mechanical environments, a visible positive trend exists.

This relationship highlights a subtle but important phenomenon: users who felt more comfortable with the robot’s limb support and movement trajectories tended to report higher feelings of safety. Conversely, participants who described minor pressure discomfort or uncertainty about the ankle interface tended to classify the system as less safe, even when the mechanical motion itself was stable. This confirms that ergonomic improvements can simultaneously influence perceived comfort and safety without changing the underlying dynamics.

Following the fuzzy logic analysis, three design domains were prioritized for immediate action: torque sufficiency, anthropometric adaptability, and perceived and physical safety. We implemented targeted mechanical and mechatronic modifications on the hip–knee parallel module and the ankle subassembly and then repeated the laboratory evaluation with the same procedures and instruments used in the pre-optimization phase. Below, we detail the engineering changes and their effect on system behavior and user/clinician ratings.

**Figure 11:**
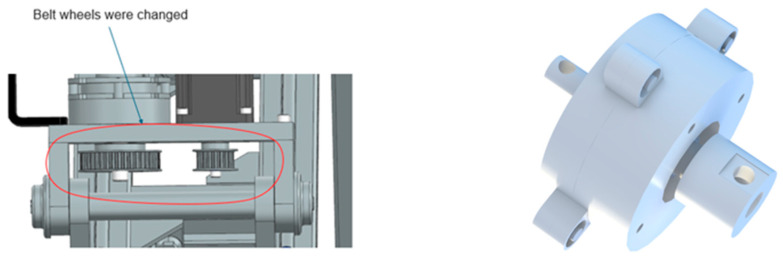
The driving axis distance and planetary gearbox.

The hip–knee axes (flexion–extension at the hip and knee, and hip abduction–adduction) were limited not by servo current but by the mechanical leverage of the drivetrain over the useful joint range. To increase available joint torque without sacrificing controllability, we combined (i) a modest increase in belt reduction where geometry allowed and (ii) the addition of a compact planetary stage on the most torque-demanding axis (see Figure 11). The design targets were set so that joint velocities remained inside the clinically desirable envelope for passive mobilization (≈5–30 °/s) while raising the stall margin for spastic or heavy limbs.

For a motor delivering a continuous torque $\tau_{m}$, the output joint torque with the overall ratio of Gand mechanical efficiency $\eta_{g}$ is

(8) $$\tau_{joint}=\eta_{g}G\tau_{m}$$

On screw-driven axes, the linear force available at the nut (lead *L*, screw efficiency $\eta_{s}$) becomes

(9) $$F_{screw}=\frac{2\pi \eta_{s}}{L}\cdot \tau_{out},\tau_{out}=\eta_{g}\cdot G\tau_{m}$$

and the maximum joint moment is then $\tau_{joint}=F_{screw}\;r_{geom}$, where *r_geom_* is the effective lever arm from the linkage synthesis. The corresponding no-load joint speed satisfies

(10) $${\dot{\theta }}_{joint}=K_{geom}\frac{{\dot{\theta }}_{m}}{G},$$

where *K_geom_* is the geometry-dependent angle gain. By trading surplus motor speed for mechanical advantage, we increased torque margins at low joint velocities typical of passive therapy while preserving smoothness and back-drivability at therapist-selected amplitudes.

Clinicians who had previously flagged “borderline torque” for imagined spastic cases reported decisively higher confidence after the transmission update. In the post-redesign questionnaire, torque sufficiency moved from a high fuzzy priority to the moderate/acceptable band, and no clinician marked “poor” in this item. Users did not perceive any loss of smoothness; the distribution of “very smooth/smooth” remained stable.

To address both actual and perceived safety, we enclosed moving screws and belts within covers. Cable routing was rationalized through low-profile carriers; all pinch zones were shielded. Emergency stop redundancy was maintained at three layers (drive, PLC, and interface), and we added hardware limit stops beyond the software soft limits.

Perceived safety ratings shifted towards “very safe,” and free-text comments referencing “visible moving parts” disappeared. In the fuzzy model, safety priority dropped below the redesign threshold and no longer dominated the decision surface.

**Figure 12:**
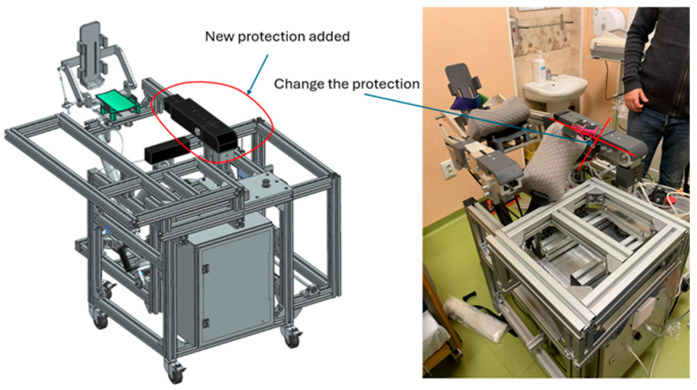
Plastic covers for perceived safety.

Comfort issues localized to the ankle brace were linked to pressure concentration at strap edges during dorsiflexion/plantarflexion. We redesigned the brace with a broader contoured cuff, dual-density padding, and a floating strap anchor that self-aligns with ankle motion to minimize shear. Hip and shank cradles received tool-less slide adjustments with engraved scales to simplify alignment for different limb lengths, and the abduction–adduction support was given a quick-release lateral offset slide to accommodate pelvic width and mild contractures (see Figure 12).

User-reported comfort and strap comfort improved; mild ankle discomfort reports decreased. Clinicians re-rated adaptability from “average/poor” to “average/good,” and setup remarks shifted from “fiddly alignment” to “routine.”

**Figure 13:**
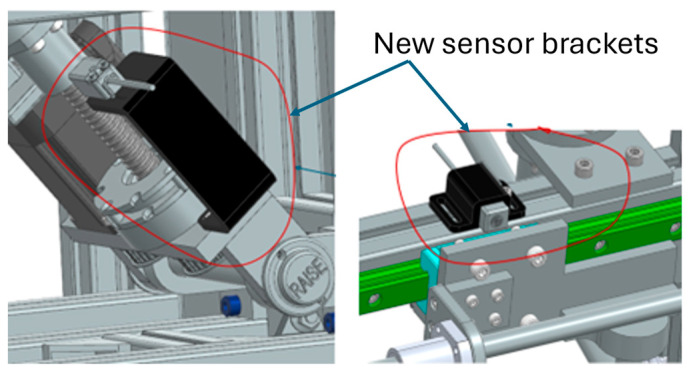
Sensor position and range limit.

We replaced flexible sensor brackets with stiffer 3D-printed glass fiber-reinforced mounts, increased the repeatability of index pulse homing, and added mechanical datum features on each axis. To reduce calibration drift, limit switch actuation cams were reprofiled and secured with captive hardware (see Figure 13).

Initial alignment became more consistent; setup/calibration effort moved from a moderate to a lower fuzzy priority. No session was interrupted by homing faults in the second evaluation round.

Although not a fuzzy priority, we addressed acoustic and thermal comfort. Belt tensioners reduced whine at mid-speeds; motor mounts gained thermal spacers to limit heat conduction to contact surfaces. Users did not report temperature-related discomfort, and background noise during operation subjectively decreased.

Following the redesign, the total transmission ratio for hip and knee actuation increased, providing higher mechanical advantage at therapeutic joint speeds (≈5–30 °/s) without driving the motors near torque limits. This configuration increased the usable output torque margin while preserving safe velocity envelopes required for passive mobilization.

Stiffening of the belt drive layout and repositioning of the motor encoders improved homing repeatability (±X° across five successive initialization trials) and reduced oscillation at the distal manipulator under low-speed motion, supporting consistent trajectory reproduction across sessions.

We repeated the full laboratory protocol after the modifications. The combined clinician–user fuzzy priority indices for torque, adaptability, and safety each dropped into the moderate or low bands, confirming that the redesign addressed the primary barriers to clinical readiness. In parallel, user-side comfort, smoothness, and trust distributions shifted rightwards, and clinician ratings of clinical utility and confidence to use with patients improved. These perceptual gains were consistent with the mechanical rationale: a higher transmission ratio increased joint torque margins at therapeutic speeds, stiffer frames and better tensioning improved tracking, and enclosure/ergonomics raised both objective safety and perceived safety.

Enclosure of moving components, improved cable management, and redundancy across PLC-based and mechanical emergency stops address standard safety expectations for bedside rehabilitation devices and were adopted following clinician feedback. Following enclosure of moving components, we performed an informal hazard screening session with senior clinicians to assess pinch point exposure, cable clearance, unintended motion under passive manipulation, and ergonomic reachability of emergency stop interfaces. These verifications preceded feasibility deployment.

Collectively, the mechanical optimization closed the loop between human-centered feedback and engineering action. The device now satisfies the torque/comfort/safety envelope required for early-stage passive mobilization and is ready to proceed to patient trials in which outcome measures (e.g., joint range, Modified Ashworth Scale, Fugl–Meyer lower extremity sub scores, and walking endurance) can be examined prospectively.

In the fuzzy inference analysis, torque sufficiency and adaptability shifted from the high-priority redesign band (*P* ≥ 0.70) to moderate values, and perceived safety dropped below the redesign threshold entirely, indicating that the optimization process materially altered the criteria caregivers considered limiting for clinical deployment.

## 4. Discussion

This study demonstrates that a parallel lower limb rehabilitation robot can be systematically optimized when the engineering process is driven by structured human feedback and a formal fuzzy inference framework. Starting from a mechanically capable baseline—comprising a parallel hip–knee module and a cable-driven ankle subsystem, actuated by servomotors under PLC supervision—the project identified specific design domains that most constrained clinical readiness. Healthy adults were enrolled as an intentional first-stage safety population, consistent with risk mitigation procedures for therapeutic mechatronics. Their normal passive stiffness and absence of pathological tone enabled the characterization of baseline limb–robot interaction without risking clonus, spastic catch, or reflex-mediated torques. Consequently, the present findings represent a lower-bound estimate of mechanical loading and cannot infer behavior in MAS ≥ 2–4 patients, in whom velocity-dependent hyper-excitability and co-contraction will require impedance-based control and real-time monitoring. When healthy users aged 25–45 and experienced clinicians appraised comfort, safety, adaptability, torque sufficiency, and setup effort, the combined and normalized scores, once mapped through carefully chosen triangular membership functions and a Mamdani rule base, converged on three redesign priorities: torque capacity, anthropometric adaptability, and perceived as well as physical safety. The coherence between qualitative comments and the fuzzy-derived crisp indices indicates that the method did not simply repackage numerical averages; rather, it emphasized those factors that clinicians weigh heavily in real practice, even when mean scores are not overtly poor.

A key limitation of the present work is that feedback was derived exclusively from neurologically healthy adults who do not exhibit spasticity, clonus, or abnormal stretch reflexes. Thus, the current torque requirements represent a lower operational bound relative to impaired populations.

The resulting mechanical interventions were grounded in first principles of actuation and structural mechanics. By trading surplus motor speed for an increased transmission ratio within the hip–knee train, the joint torque margin was raised in the clinically relevant, low-velocity range of passive mobilization, without introducing perceptible loss of smoothness or back-drivability. Stiffness enhancements to the frame and belt guidance mitigated compliance and backlash, improving motion fidelity during reversals and at end ranges, which participants recognized as greater smoothness and control. Comprehensive covers for lead screws and belts, together with rationalized cable routes and redundant emergency stops, simultaneously reduced objective risk and the visual cues that trigger perceptions of danger. Ergonomic refinement of the ankle interface and quick, tool-less limb-length adjustments addressed the discomfort and alignment variability that commonly impair tolerability and throughput. These targeted actions shifted post-test subjective distributions toward “very safe,” “very smooth,” and “useful,” while the fuzzy priority indices for torque, adaptability, and safety fell from the high to the moderate or low bands, indicating that the optimization attained its immediate objectives.

In the supine position, stiffness and force transmission dominate overweight bearing considerations, and the capacity to reproduce constrained, biomimetic joint trajectories with low deflection is advantageous. Parallel architecture provides this stiffness with compact envelopes and distributes loads efficiently across multiple limbs of the mechanism. The cable-driven ankle unit complements the parallel hip–knee chain by separating distal degrees of freedom and allowing compliant coupling that reduces small misalignments.

The increased transmission ratios, combined with safety-bounded joint speeds, were selected specifically in anticipation of mobilizing limbs with elevated tone. Clinicians emphasized that torque margins are critical during involuntary resistance, and these considerations directly motivated the actuation changes reported here.

The ergonomic redesign of the ankle module and the tool-less anthropometric adjustments were motivated by expected asymmetry and mild contracture in stroke survivors, and we anticipate that these features will become more critical in clinical populations than in healthy users.

The fuzzy logic layer added value in three specific ways. First, it preserved interpretability for clinical stakeholders; inputs labeled as comfort, safety, torque sufficiency, adaptability, and setup effort retained intuitive linguistic meaning, and the rule base encoded expert priorities explicitly (for example, low safety implies very high redesign urgency, regardless of other favorable ratings). Second, it was robust to ordinal and mildly non-Gaussian questionnaire data. Because membership degrees aggregate soft evidence rather than relying on strict parametric assumptions, the inference remained stable in the face of small sample heterogeneity that is typical of feasibility studies. Third, defuzzified crisp indices produced an actionable ranking that mapped directly onto engineering choices, facilitating resource allocation without obscuring the underlying trade-offs. The radar representation and correlation structure buttressed these conclusions, revealing expected couplings between torque and adaptability and between comfort and safety, which explains why ergonomic changes can improve perceived safety, even when actuator limits are unchanged.

The current fuzzy framework is modular and will be recalibrated once patient-specific metrics (e.g., spasticity-related discomfort, involuntary co-contraction, and interaction-torque peaks) are collected. This data will allow for an adjustment of rule weights and membership functions to reflect real patient priorities during active or active-assisted therapy.

Several methodological considerations constrain the conclusions that can be drawn from this study. The cohort comprised healthy adults rather than patients with neurological impairments, which constrains external validity for spasticity, clonus, sensory hypersensitivity, and joint contracture—conditions that often dictate real-world torque requirements and interface tolerances. The sessions were purely passive; consequently, neither patient effort modulation nor reflexive responses were engaged, and the dynamic interaction terms that would appear under active participation were not estimated. The questionnaires, while structured and mapped to consistent scales, remained subjective by design. Although the fuzzy framework mitigated some of the limitations of ordinal data, it cannot substitute for objective physiological measures such as electromyography, joint contact pressure mapping, or precise end-effector force tracking during high-tone episodes.

These limitations indicate a clear path for future work. The dynamic model already formulated in joint coordinates provides a basis for adaptive, assist-as-needed control that combines computed torque feedforward with impedance modulation. Two directions appear most relevant for early neurorehabilitation. First, admittance control can adjust trajectory compliance based on measured interaction torques, using estimated human impedance to tune the robot’s apparent stiffness in real time. Second, progressive assistance can be driven by performance indicators—such as tracking errors, interaction energy, or simple clinical rules—so that training evolves from passive to increasingly active participation. Both strategies are compatible with the existing PLC–drive architecture. Torque or force observers at the drive level can be made available to a supervisory layer, where the C# interface would expose therapist-friendly targets rather than raw control gains.

The dynamic model and PLC-based architecture are compatible with impedance or admittance-based assist-as-needed strategies, facilitating progression from passive to interactive control once patient trials commence.

From a translational standpoint, the workflow adopted here—collecting questionnaires, merging clinician and user feedback through a fuzzy logic layer, implementing focused mechanical updates, and validating with the same instruments—offers a practical, resource-efficient template for iterative development. Because the rule base is explicit, it can be refined during patient trials; for example, by increasing the weight of safety in acute settings or prioritizing setup time in busy outpatient clinics. The same framework can also incorporate workload or throughput metrics, enabling engineering choices to address not only patient comfort and safety but also operational efficiency, which ultimately drives adoption. It is important to emphasize that the present work represents a preparatory stage for forthcoming clinical evaluation. The primary objective of this study was not to assess patient outcomes directly but to optimize the rehabilitation robot based on structured clinician-derived requirements and iterative engineering refinements. By integrating expert feedback through a transparent fuzzy logic framework and validating subsequent mechanical and control improvements, we ensured that the redesigned platform aligns with safety, usability, and therapeutic expectations observed in early-stage rehabilitation. With the optimized device now meeting these clinician-informed criteria, the next phase will involve conducting patient trials to systematically evaluate functional outcomes, user tolerability, and real-world clinical performance. This progression—from clinician-centered optimization to patient-centered validation—establishes a coherent translational pathway for advancing the system toward routine clinical adoption.

Clinicians estimated a 5–10 min mounting and anthropometric-fitting window, which aligns with typical orthotic preparation time for passive lower limb mobilization in inpatient settings, suggesting that workflow burden is compatible with routine care.

The study illustrates how human-centered feedback, when embedded in a transparent fuzzy logic framework and connected tightly to actuation and structural redesign, can advance a rehabilitation robot from a technically functional prototype to a clinically plausible platform. The optimized device now aligns with early-stage therapeutic needs—safe passive mobilization with sufficient torque margins, ergonomic interfaces that accommodate anthropometric variability, and reliable, deterministic control—and it is positioned for patient trials where objective functional outcomes, safety events, and workflow metrics can be measured rigorously. Although the redesigned actuation increased low-velocity torque margins, patients exhibiting severe tone elevations (e.g., MAS ≥ 3) may still generate resistance above comfortable passive mobilization thresholds. Such cases will require adaptive impedance/admittance control rather than pure position enforcement and may necessitate staged progression.

## 5. Conclusions

This work presents a framework for advancing a parallel robotic system from a functional prototype to a clinically plausible platform for early-stage lower limb rehabilitation. In this work, “clinical-trial readiness” refers to mechanical reliability, passive-mode safety, and clinician usability that justify transition to feasibility studies. No inference regarding therapeutic superiority can be made until controlled patient trials are completed.

By combining detailed kinematic modeling, structured user and clinician evaluation, and a Mamdani-type fuzzy logic model, the study identified torque sufficiency, anthropometric adaptability, and physical and perceived safety as the principal barriers to clinical deployment. The iterative engineering refinements—targeted toward mechanical transmission, structural stiffness, enclosure of moving components, and ergonomic redesign of patient interfaces—directly addressed these limitations and resulted in measurable improvements across user comfort, movement smoothness, trust, and clinician-rated usability. The post-optimization fuzzy indices confirmed that previously critical aspects had shifted into acceptable or low-priority domains, validating the effectiveness of the feedback-driven redesign process. Beyond improving this specific device, the study demonstrates a practical methodology for integrating clinician and user perspectives into the development cycle of rehabilitation robots, especially when sample sizes are modest and feedback is inherently qualitative. The resulting LegUp platform now aligns with the mechanical and safety requirements for early-stage passive mobilization and is prepared for clinical trials that will evaluate functional outcomes, patient tolerability, and workflow integration in real rehabilitation settings. This transition from prototype refinement to patient-focused validation establishes a clear translational path toward future clinical adoption.

## Figures and Tables

**Table 1:** User questionnaire.

Item No.	Questionnaire Item	Response Scale
1	How clearly was the procedure explained to you before beginning the session?	Very clear/Clear/Neutral/ Unclear/Very unclear
2	How easy was it to position yourself inside the robot?	Very easy/Easy/Neutral/ Difficult/Very difficult
3	Did you feel physically well supported during positioning?	Yes, completely/Mostly/Neutral/ Not really/Not at all
4	How comfortable did you feel during hip and knee movement?	Very comfortable/Comfortable/ Neutral/Uncomfortable/ Very uncomfortable
5	Did you feel pain or discomfort during any movement?	No pain/Mild discomfort/ Moderate discomfort/Severe pain
6	How safe did you feel while interacting with the robotic system?	Very safe/Safe/Neutral/Unsafe/ Very unsafe
7	How natural did the hip–knee motion feel?	Very natural/Natural/Neutral/ Unnatural/Very unnatural
8	How natural did the ankle motion feel?	Very natural/Natural/Neutral/ Unnatural/Very unnatural
9	Did the movements feel synchronized as a whole?	Completely synchronized/Mostly synchronized/Neutral/Slightly asynchronous/Very asynchronous
10	Did you experience physical fatigue during or after the session?	Not tiring/Slightly tiring/ Moderately tiring/Very tiring
11	Did your limb feel properly stabilized throughout the session?	Completely stable/Mostly stable/Neutral/Unstable/ Very unstable
12	How useful do you consider the ankle module to be?	1 = Not useful at all/2 = Not useful/3 = Somehow useful/4 = Useful/5 = Very useful
13	How well did the system fit your body dimensions?	Excellent fit/Good fit/ Acceptable/Poor fit/Very poor fit
14	Did you feel in control of the stop or exit options, if needed?	Yes, completely/Yes, mostly/ Not sure/Slightly/Not at all
15	How would you rate the overall experience?	1 = Very poor … 5 = Excellent
16	Would you use a system like this again in a real rehabilitation setting?	Definitely yes/Probably yes/ Not sure/Probably not/Definitely not
17	What did you appreciate most about the robotic system?	Open-ended
18	What aspects do you think should be improved?	Open-ended

**Table 2:** Clinician questionnaire.

Item No.	Questionnaire Item	Response Scale
1	How easy was the setup procedure for this device?	Very easy/Easy/Neutral/Difficult/Very difficult
2	How long would it take to position a typical patient?	<2 min/2–5 min/5–10 min/>10 min
3	How adaptable is the system to patients of different sizes?	Excellent adaptability/Good/Moderate/Poor/Very poor
4	How clinically relevant is the movement supported by this robot?	Excellent/Good/Acceptable/Limited/Irrelevant
5	How would you assess patient comfort during passive mobilization?	Excellent/Good/Average/Poor/Very poor
6	How safe do you consider the robot for use in a clinical setting?	Very safe/Safe/Neutral/Unsafe/Very unsafe
7	Is the torque capacity sufficient for patients with spasticity or muscle tone?	Fully sufficient/Mostly sufficient/Borderline/ Insufficient/Strongly insufficient
8	How useful is the ankle module for clinical goals?	1 = Not useful at all/2 = Not useful/3 = Somehow useful/4 = Useful/5 = Very useful
9	How intuitive and efficient is the software interface?	Very intuitive/Clear/Neutral/Confusing/Very confusing
10	How much does the system affect clinician workload?	Greatly reduces/Slightly reduces/Neutral/Slightly increases/Greatly increases
11	How likely would you be to integrate this device in a real therapy plan?	Very likely/Likely/Possible/Unlikely/Impossible
12	Global evaluation of the system’s rehabilitation potential	1 = Very poor/2 = Poor/3 = Good/4 = Very good/5 = Excellent
13	Which aspects do you most appreciate from a clinical perspective?	Open-ended
14	Which elements would need to be improved for clinical adoption?	Open-ended

**Table 3:** Descriptive statistics of clinician and user questionnaire responses.

Group	Questionnaire Item	Mean (M)	Standard Deviation (SD)
Clinicians	Torque Adequacy	4.60	0.55
	Adaptability	4.80	0.45
	Usability	4.80	0.45
	Safety	4.80	0.45
Users	Comfort	4.57	0.51
(7 Patients)	Smoothness of Movement	4.43	0.73
	Trust in the Robot?	4.86	0.35

## Data Availability

Data is contained within the article.

## Appendix A

**Table A1:** End-user questionnaire descriptive statistics.

Items (Users)	Mean	SD	Interpretation
Comfort during passive movement	4.4	0.51	High comfort
Perceived safety/trust in system	3.8	0.72	Moderately high trust
Ease of mounting/positioning	3.5	0.80	Mild ergonomic strain
Torque sufficiency (ability to move leg easily)	3.2	0.64	Borderline adequate
Adaptability to different limb sizes	3.3	0.69	Moderate concern
Overall acceptance	4.1	0.58	Positive experience

**Table A2:** Clinician questionnaire descriptive statistics.

Items (Clinicians)	Mean	SD	Interpretation
Safety adequacy for passive ROM	3.4	0.81	Mid-level concern
Torque adequacy for mobilization	2.9	0.55	Insufficient torque
Adaptability/anthropometric fitting	3.0	0.51	Needs redesign
Clinical applicability/usefulness	3.5	0.6	Cautious acceptance
Workflow burden (time/supervision)	3.2	0.66	Requires refinement
Readiness for patient exposure	3.1	0.48	Borderline feasible

**Table A3:** Fuzzy logic priority aggregation table.

Domain	User Score (0–1 Normalized)	Clinician Score (0–1 Normalized)	Weighted Score (0.6 Clin, 0.4 User)	Mamdani Output P	Priority
Torque Sufficiency	0.64	0.48	0.54	0.82	High redesign
Adaptability	0.66	0.50	0.56	0.78	High redesign
Safety and Trust	0.76	0.68	0.72	0.65	Medium priority
Comfort	0.88	0.74	0.80	0.42	Low priority
Workflow Impact	0.70	0.63	0.67	0.57	Moderate
